# The real‐world applicability of the 2023 international myelin oligodendrocyte glycoprotein antibody‐associated disease criteria in a Latin American cohort

**DOI:** 10.1111/ene.16445

**Published:** 2024-09-17

**Authors:** Edgar Carnero Contentti, Claudia Pestchanker, Ethel Ciampi, Sheila Castro Suarez, Cesar Caparo Zamalloa, Vanesa Daccach Marques, Katharina Messias, José Ignacio Gortari, Verónica Tkachuk, Berenice Silva, Carolina Mainella, Saúl Reyes, Jaime Toro, Juan Rodriguez, Edgar Correa‐Diaz, Juan I. Rojas, Friedemann Paul

**Affiliations:** ^1^ Neuroimmunology Unit, Department of Neurosciences Hospital Aleman Buenos Aires Argentina; ^2^ Neurology Department Dr. Ramón Carrillo Central Hospital San Luis Argentina; ^3^ Neurology Department Hospital Dr. Sótero del Río y Universidad Católica de Chile Santiago Chile; ^4^ Basic Research Center in Dementia and Central Nervous System Demyelinating Diseases Instituto Nacional de Ciencias Neurológicas Lima Peru; ^5^ Hospital das Clínicas da Faculdade de Medicina de Ribeirão Preto Universidade de São Paulo São Paulo Brazil; ^6^ Neurology private practice Pereira Colombia; ^7^ Neurology Department Hospital de Clínicas Buenos Aires Argentina; ^8^ Neurology Department Hospital Italiano de Buenos Aires Buenos Aires Argentina; ^9^ Neurology Department Hospital Español de Rosario Santa Fe Argentina; ^10^ Neurology Department Fundación Santa Fe de Bogotá Bogotá Colombia; ^11^ School of Medicine Universidad de los Andes Bogotá Colombia; ^12^ Blizard Institute, Barts and the London School of Medicine and Dentistry Queen Mary University of London London UK; ^13^ Department of Neurology Hospital Carlos Andrade Marín Quito Ecuador; ^14^ Pontificia Universidad Católica del Ecuador Quito Ecuador; ^15^ Neurology Department Centro de Esclerosis Múltiple de Buenos Aires Buenos Aires Argentina; ^16^ NeuroCure Clinical Research Center Charité—Universitätsmedizin Berlin, corporate member of Freie Universität Berlin, Humboldt‐Universität zu Berlin and Berlin Institute of Health Berlin Germany; ^17^ Experimental and Clinical Research Center Max Delbrueck Center for Molecular Medicine and Charité—Universitätsmedizin Berlin Berlin Germany

**Keywords:** diagnostic criteria, Latin America, MOGAD, optic neuritis, transverse myelitis

## Abstract

**Background and Purpose:**

The diagnostic criteria for myelin oligodendrocyte glycoprotein antibody (MOG‐IgG)‐associated disease (MOGAD) were published in 2023. We aimed to determine the performance of the new criteria in Latin American (LATAM) patients compared with the 2018 criteria and explore the significance of MOG–IgG titers in diagnosis.

**Methods:**

We retrospectively reviewed the medical records of LATAM (Argentina, Chile, Brazil, Peru, Ecuador, and Colombia) adult patients with one clinical MOGAD event and MOG‐IgG positivity confirmed by cell‐based assay. Both 2018 and 2023 MOGAD criteria were applied, calculating diagnostic performance indicators.

**Results:**

Among 171 patients (predominantly females, mean age at first attack = 34.1 years, mean disease duration = 4.5 years), 98.2% patients met the 2018 criteria, and of those who did not fulfill diagnostic criteria (*n* = 3), all tested positive for MOG‐IgG (one low‐positive and two without reported titer). Additionally, 144 (84.2%) patients met the 2023 criteria, of whom 57 (39.5%) had MOG‐IgG+ titer information (19 clearly positive and 38 low‐positive), whereas 87 (60.5%) patients had no MOG‐IgG titer. All 144 patients met diagnostic supporting criteria. The remaining 27 patients did not meet the 2023 MOGAD criteria due to low MOG‐IgG (*n* = 12) or lack of titer antibody access (*n* = 15), associated with the absence of supporting criteria. The 2023 MOGAD criteria showed a sensitivity of 86% (95% confidence interval = 0.80–0.91) and specificity of 100% compared to the 2018 criteria.

**Conclusions:**

These findings support the diagnostic utility of the 2023 MOGAD criteria in an LATAM cohort in real‐world practice, despite limited access to MOG‐IgG titration.

## INTRODUCTION

Myelin oligodendrocyte glycoprotein antibody (MOG‐IgG)‐associated disease (MOGAD) is a rare and recently defined demyelinating disorder of the central nervous system (CNS), characterized by relapses of optic neuritis (ON), transverse myelitis (TM), and brainstem/brain impairment with a rapidly evolving clinical spectrum [1, 2]. Currently, a significant overlap of clinical and neuroradiological findings with aquaporin‐4 antibody (AQP4‐IgG) neuromyelitis optica spectrum disorder (NMOSD) and multiple sclerosis (MS) are commonly observed in clinical practice [1, 2, 3, 4, 5]. However, MOGAD is considered a different entity from AQP4‐IgG NMOSD and MS [1, 2, 3, 4, 5]. Recently, this disease‐specific antibody that binds MOG has been identified based on new generation cell‐based assays (CBAs), leading initially (in 2018) to the publication of two “not formal” sets of criteria based on MOGAD international recommendations for diagnosis and antibody testing [6] and a single referral center (Mayo Clinic) [7]. Most recently, the definition and classification of MOGAD was published by an international panel of experts who described the 2023 proposed diagnostic criteria for this entity [4]. This International MOGAD Panel has highlighted three main points to reach diagnosis: (i) core clinical demyelinating events and supporting clinical or magnetic resonance imaging (MRI) features, (ii) MOG‐IgG and their titers, and (iii) exclusion of alternative diagnoses. Thus, the 2023 diagnostic criteria have emphasized the serostatus and clinical implications of MOG‐IgG plus typical or suggestive MRI lesions, reflecting broader MOGAD phenotypes, to facilitate earlier and more accurate diagnosis [4]. Notably, if MOG‐IgG titers are low‐positive or positive without reported titer or negative but with clearly positive cerebrospinal fluid (CSF) MOG‐IgG, supporting clinical or MRI criteria must be met to establish an MOGAD diagnosis. It is important to notice that the availability for MOG‐IgG testing has been reported to be <42% in lower income or lower resource countries like Latin American (LATAM) countries [8]. Thus, the access to MOGAD care and cost of recommended assays (including antibody titers) are a limitation in fulfilling diagnostic criteria, leading to evident challenges in achieving an early, accurate, and definitive diagnosis in this population. This issue is well recognized, as patients may exhibit clinical and imaging features consistent with MOGAD but may not have detectable MOG‐IgG or they may live in countries where reliable MOG‐IgG testing is unavailable.

The 2023 MOGAD criteria have shown a good performance in Asian [9], North American [10, 11], and European [12] populations, demonstrating the utility of these new criteria. However, as there have been no studies assessing the 2023 MOGAD criteria application in LATAM populations, our goal was to determine whether these new criteria enhance the diagnostic rate and how the absence of MOG‐IgG titers impacts in clinical practice.

## METHODS

We retrospectively reviewed the medical records at first attack of consecutive adult patients (≥18 years of age) with at least one core demyelinating clinical MOGAD event at onset or during follow‐up: TM, ON, acute disseminated encephalomyelitis (ADEM), cerebral monofocal or polyfocal deficits, brainstem or cerebellar deficits, and/or cerebral cortical encephalitis, associated with MOG‐IgG by CBA positivity in serum or CSF tests. To mitigate selection bias, neurologists had to register all patients seen in clinical practice with phenotypes suggestive of NMOSD/MOGAD and they were asked to submit information on any patient with at least one core clinical demyelinating event of MOGAD plus MOG‐IgG+. We included all consecutive patients seen from January 2018 to December 2023 at specialized centers in Argentina (*n* = 35), Chile (*n* = 53), Brazil (*n* = 33), Peru (*n* = 37), Ecuador (*n* = 3), and Colombia (*n* = 10). Data on gender, ethnicity, age, and symptoms at onset, MOG‐IgG testing setting, typical lesions on MRI, and time of starting immunosuppressive therapy were collected. We classified patients according to four major ethnicity groups: mixed (people of mixed European and Amerindian ancestry living in the region of Latin America), Caucasian (individuals of European descent), Afro‐descendant (individuals of mixed native American and African descent), and Asian (a person having origins in any of the original peoples of the Far East, Southeast Asia, or the Indian subcontinent) as described previously [12].

MOG‐IgG and AQP4‐IgG status was measured using live or fixed CBA in all included patients, and repeated values (if applicable) were analyzed [13, 14]. Retesting was performed as required by the treating neurologist. Positivity for serum MOG‐IgG was divided into clearly positive, low‐positive, or positive without reported titer, as described in the 2023 MOGAD criteria [4].

As shown in Table 1, MOGAD diagnosis was reached if patients met the 2018 and/or 2023 MOGAD criteria in accordance with Jarius et al. [6] and Wingerchuk et al. [4], respectively. Notably, all patients (100%) met the 2018 Mayo Clinic MOGAD criteria [7].

**TABLE 1:** Comparison between 2018 and 2023 MOGAD diagnostic criteria.

2018 Mayo Clinic criteria [7] (meet all of the following)	2018 criteria [6] (meet all of the following)	2023 criteria [4] (meet A, B, and C and supporting criteria if needed)
Clinical findings: any of the following presentations: ADEM ON, including CRION Transverse myelitis (i.e., LETM or STM) Brain or brainstem syndrome compatible with demyelination Any combination of the above	Monophasic or relapsing acute ON Myelitis Brainstem encephalitis or encephalitis Any combination of these syndromes	(A) Core clinical demyelinating event ON Myelitis ADEM Cerebral monofocal or polyfocal deficits Brainstem or cerebellar deficits Cerebral cortical encephalitis often with seizures
Serum positive for MOG‐IgG by cell‐based assay (in absence of serum, positivity in CSF would allow fulfillment of lab criteria)	Seropositivity for MOG‐IgG (cell‐based assay employing full‐length human MOG as target antigen)	(B) Positive MOG‐IgG test (serum cell‐based assay) Clearly positive: no additional supporting features required Low‐positive, positive without reported titer, or negative but CSF positive: requires AQP4‐IgG seronegative AND ≥1 supporting clinical or MRI feature
	MRI or electrophysiological (visual evoked potentials in patients with isolated ON) findings compatible with CNS demyelination	Supporting clinical or MRI features ON ○ Bilateral simultaneous clinical involvement ○ Longitudinal optic nerve involvement (>50% length of the optic nerve) ○ Perineural optic sheath enhancement ○ Optic disc edema Myelitis ○ Longitudinally extensive myelitis ○ Central cord lesion or H sign ○ Conus lesion Brain, brainstem, or cerebral syndrome ○ Multiple ill‐defined T2‐hyperintense lesions in supratentorial and often infratentorial white matter ○ Deep grey matter involvement ○ Ill‐defined T2‐hyperintensity involving pons, middle cerebellar peduncle, or medulla ○ Cortical lesion with or without lesional and overlying meningeal enhancement
Exclusion of alternative diagnosis	(C) If a red flag is present, they should receive a label of possible MOGAD	(C) Exclusion of better diagnoses including multiple sclerosis

*Source*: Adapted from López‐Chiriboga et al. [7], Jarius et al. [6], and Banwell et al. [2].

Abbreviations: ADEM, acute disseminated encephalomyelitis; AQP4‐IgG, aquaporin‐4 antibody; CNS, central nervous system; CRION, chronic relapsing inflammatory optic neuropathy; CSF, cerebrospinal fluid; LETM, longitudinal extensive transverse myelitis; MOGAD, myelin oligodendrocyte glycoprotein antibody (MOG‐IgG)‐associated disease; MOG‐IgG, myelin oligodendrocyte glycoprotein antibody; MRI, magnetic resonance imaging; ON, optic neuritis; STM, short transverse myelitis.

All patients and MRI scans supporting clinical or MRI features were evaluated by at least one of the authors (neurologists/neuroimmunologists) and one neuroradiologist (all of them with expertise in demyelinating diseases). Although there was no standardized orbit, brain, and spinal cord conventional MRI protocol among centers, brain scans included T2‐weighted imaging, fluid‐attenuated inversion recovery, gadolinium‐enhanced T1‐weighted imaging, and/or diffusion‐weighted imaging; orbital scans included fat‐suppression, and spinal cord included short tau inversion recovery. Thus, all available MRIs with and without contrast at the time of the diagnosis (during an attack within 30 days of symptom presentation) were reviewed. Additionally, no standardized clinical or ophthalmological assessments were performed. Serum samples were determined in different laboratories according to each participating patient/center, and noncentralized determinations were obtained, reflecting real‐world evidence of clinical practice in a realistic setting. Exclusion of better diagnoses or alternative diagnoses including MS and NMOSD was based on the judgment of each clinical neurologist. Patients with insufficient clinical or serologic data required for the minimal dataset were excluded.

To ensure consistent data collection, a dedicated web‐based platform was created to investigate MOGAD diagnosis, and researchers were requested to register and share relevant patient data for the study. Because our clinical practice has been based on the 2018 recommendations until recently, we found it reasonable to compare the 2023 criteria with the 2018 diagnostic recommendations; therefore, both the 2018 international diagnostic recommendations for MOG‐encephalomyelitis [6] and the 2023 MOGAD diagnostic criteria [4] were retrospectively applied to our entire cohort at first attack and during the follow‐up period to evaluate the diagnostic performance (Table 1).

Each participating center obtained approval from an ethics committee, and written or oral informed consent (according to each committee, if necessary) for the use of their anonymized data for research purposes was obtained from all participants before data collection.

### Statistical analysis

Statistical analyses were conducted using SPSS v22 and GraphPad Prism 8 software. Continuous data for group comparisons were assessed using Student *t*‐test or Mann–Whitney *U*‐test, whereas categorical data were analyzed using the chi‐squared test or Fisher exact test, as appropriate. Results were reported as proportion, mean, SD, and median.

True positive (TP), true negative (TN), false positive (FP), and false negative (FN) were defined as follows. TP was defined as the number/proportion of patients who met the 2023 criteria, had clinical features and were MOG‐IgG+ as determined by neurologists, and met the 2018 criteria. TN was defined as the number/proportion of patients who did not meet the 2023 criteria, were false positive for MOG‐IgG, and did not meet the 2018 criteria. FP was defined as the number/proportion of patients who met the 2023 criteria, were false positive for MOG‐IgG, and did not meet the 2018 criteria. FN was defined as the number/proportion of patients who did not meet the 2023 criteria, had clinical features and were MOG‐IgG+ as determined by neurologists, and met the 2018 criteria.

Sensitivity (TP/TP + FN), specificity (TN/FP + TN), positive predictive values (PPVs; PPV = TP/TP + FP), and negative predictive values (NPVs; NPV = TN/TN + FN) were calculated. A significance level of 5% (*p* < 0.05) was set for all analyses.

## RESULTS

A total of 190 patients from six LATAM countries were collected, of whom 171 were included in the analysis. Nineteen patients with an initial attack before 18 years of age or with insufficient data were excluded.

### General characteristics

**FIGURE 1:**
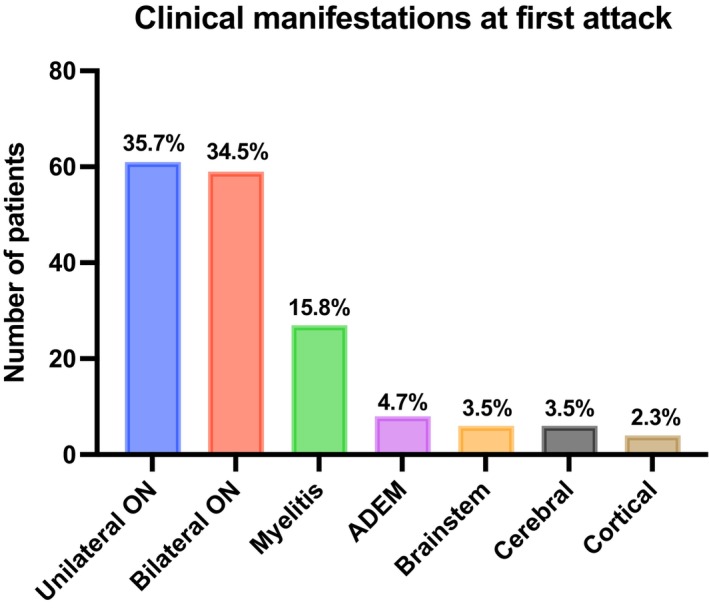
First core clinical demyelinating event. ADEM, acute disseminated encephalomyelitis.

**FIGURE 2:**
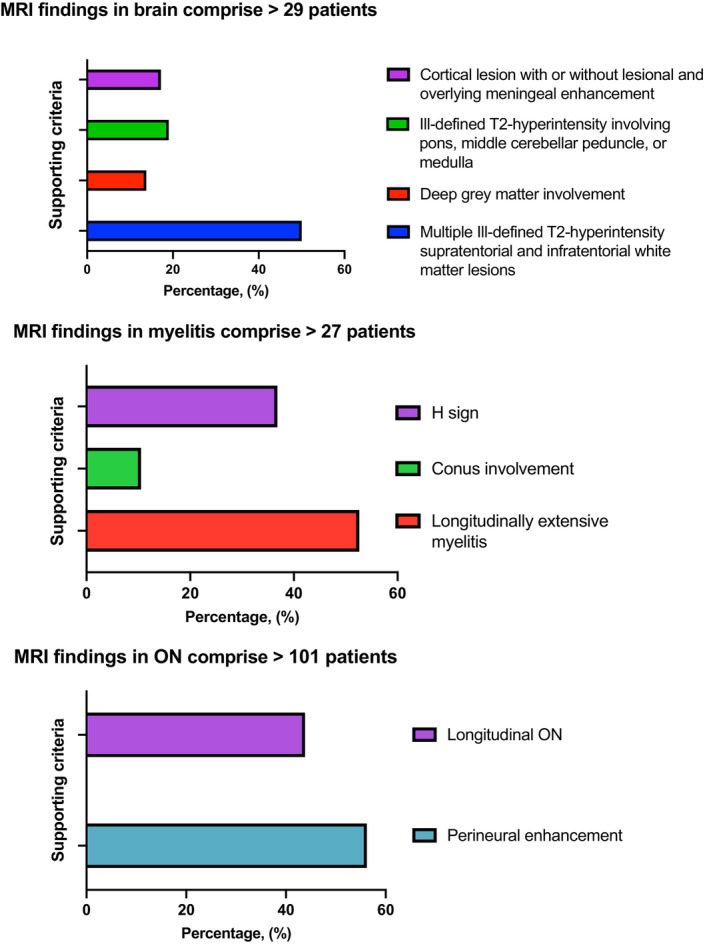
Magnetic resonance imaging (MRI) findings. MRI findings from the initial attack were analyzed in 157 MRI studies conducted on 131 patients within 30 days of the onset of clinical symptoms. ON, optic neuritis.

As shown in Table 2, there was a slight predominance of females (59.1%), with a mean age at first attack of 34.1 (±12.8) years and a mean disease duration of 4.5 (±5.8) years. The Caucasian population (55.6%) was the most common ethnicity, followed by mixed (40.9%). MOG‐IgG test results were as follows: clearly positive, *n* = 19 (11.1%); low‐positive, *n* = 48 (28.1%); and positive without reported titer, *n* = 103 (60.2%). Assays performed for serum MOG‐IgG were as follows: fixed CBA, *n* = 120 (70.1%); live CBA, *n* = 3 (1.7%); and not reported CBA, *n* = 48 (28.1%). A repeated serum MOG‐IgG test was obtained in 16 patients, with 81.2% of them being positive without a reported titer. Of note, many LATAM laboratories that perform MOG‐IgG and AQP4‐IgG testing by CBA do not report whether the assay is based on live or fixed cells. Interestingly, MOG‐IgG test in CSF was not obtained in any included LATAM patient. AQP4‐IgG test was performed in all included patients, with none of them testing positive. MOG‐IgG test was conducted during an attack and before acute treatment in 90 (52.4%) patients. As expected, isolated ON (*n* = 97; unilateral ON, *n* = 52; bilateral ON, *n* = 45) was the most common manifestation at disease onset, followed by myelitis (*n* = 26), as illustrated in Figure 1. Additionally, 131 (76.6%) patients had MRI available at the time of the first attack (performed before 30 days from the beginning of symptoms). In patients with ON, the most common MRI finding was perineural enhancement (52.2%), whereas patients with TM showed longitudinal extensive TM lesions (52.6%) as the most frequent compromise. Additionally, in cases of brain involvement, multiple ill‐defined T2 hyperintense supra‐ and infratentorial white matter lesions were observed. Frequency of MRI lesions is illustrated in Figure 2.

**TABLE 2:** Demographic and MOG‐IgG testing information of the studied cohort.

Enrolled patients, *N*	171
Current age, years	38.6 (±13.1)
Age at onset, years	34.1 (±12.8)
Mean follow‐up duration, years	4.5 (±5.8)
Median, years (IQR)	2 (1–5)
Female	100 (59.1)
Ethnicity	
Mixed	70 (40.9)
Caucasian	95 (55.6)
Afro‐descendant	2 (1.2)
Asian	1 (0.6)
Other	3 (1.7)
Countries	
Argentina	35 (20.4)
Brazil	33 (19.3)
Chile	53 (30.9)
Peru	37 (21.6)
Ecuador	3 (1.7)
Colombia	10 (5.8)
Serum MOG‐IgG test at the time of first attack	
Clearly positive	19 (11.1)
Low‐positive	48 (28.1)
Positive without reported titer	103 (60.2)
Assays performed for serum MOG‐IgG	
Live CBA	3 (1.7)
Fixed CBA	120 (70.1)
CBA not reported	48 (28.1)
Repeat serum MOG‐IgG test, *n* = 16^a^	
Clearly positive	2 (12.5)^a^
Low‐positive	1 (6.2)
Positive without reported titer	13 (81.2)
Assays performed for repeat serum MOG‐IgG	
Live CBA	14 (87.5)^a^
Fixed CBA	1 (6.2)
CBA not reported	1 (6.2)
Assays performed for serum AQP4‐IgG	
Live CBA	39 (22.4)
Fixed CBA	120 (70.5)
Tissue‐based IIF	7 (4.1)
Unknown	5 (3)
Relationship between MOG‐IgG testing and acute treatment	
Before acute treatment	90 (52.4)
After acute treatment	81 (47.7)

*Note*: Data are presented as mean (±SD), median (IQR), or *n* (%). Abbreviations: AQP4‐IgG, aquaporin‐4 antibody; CBA, cell‐based assay; IIF, indirect immunofluorescence; IQR, interquartile range; MOG‐IgG, myelin oligodendrocyte glycoprotein antibody.

^a^ Fifteen patients were also tested in a second sample, and one patient was tested three times.

Among supporting criteria for ON, the most frequent finding was bilateral ON (36.4%), followed by perineural enhancement (30.8%), as shown in Table 3.

**TABLE 3:** Frequency of supporting criteria in patients meeting 2023 MOGAD diagnostic criteria based on clinical manifestations and serostatus.

Optic neuritis					
Total support criteria	Perineural enhancement	Longitudinal ON	Disc edema	Bilateral optic neuritis	MOG‐IgG serostatus
40	13 (32.5%)	9 (22.5%)	6 (15%)	12 (30%)	Low‐positive (*n* = 29)
108	34 (31.4%)	28 (25.9%)	3 (2.7%)	43 (39.8%)	No titer (*n* = 61)
14	3 (21.4%)	2 (14.2%)	5 (35.7%)	4 (28.5%)	Clearly positive (*n* = 11)
162	50 (30.8%)	39 (24%)	14 (8.6%)	59 (36.4%)	Total (*n* = 101)

Myelitis				
Total support criteria	Longitudinal involvement	Conus involvement	Central cord or H sign	MOG‐IgG serostatus
9	6 (66.6%)	2 (22.2%)	1 (11.1%)	Low‐positive (*n* = 7)
32	18 (56.2%)	1 (3.1%)	13 (40.6%)	No titer (*n* = 24)
9	7 (77.7%)	0 (0%)	2 (22.2%)	Clearly positive (*n* = 7)
50	31 (62%)	3 (6%)	16 (32%)	Total (*n* = 38)

Brain, brainstem, or cerebral syndrome					
Total support criteria	Multiple ill‐defined T2‐hyperintense lesions in supratentorial and often infratentorial white matter	Deep grey matter involvement	Ill‐defined T2‐hyperintensity involving pons, middle cerebellar peduncle, or medulla	Cortical lesion with or without lesional and overlying meningeal enhancement	MOG‐IgG serostatus
7	4 (57.1%)	1 (14.2%)	1 (14.2%)	1 (14.2%)	Low‐positive (*n* = 6)
24	14 (58.3%)	2 (8.3%)	3 (12.5%)	3 (12.5%)	No titer (*n* = 20)
1	1 (100%)	0 (0%)	0 (0%)	0 (0%)	Clearly positive (*n* = 1)
32	19 (59.4%)	3 (9.4%)	4 (12.5%)	4 (12.5%)	Total (*n* = 27)

*Note*: Proportion was calculated over total supporting criteria.

Abbreviations: MOGAD, myelin oligodendrocyte glycoprotein antibody (MOG‐IgG)‐associated disease; MOG‐IgG, myelin oligodendrocyte glycoprotein antibody; ON, optic neuritis.

### MOGAD diagnostic performance

#### Applicability of 2018 criteria

**FIGURE 3:**
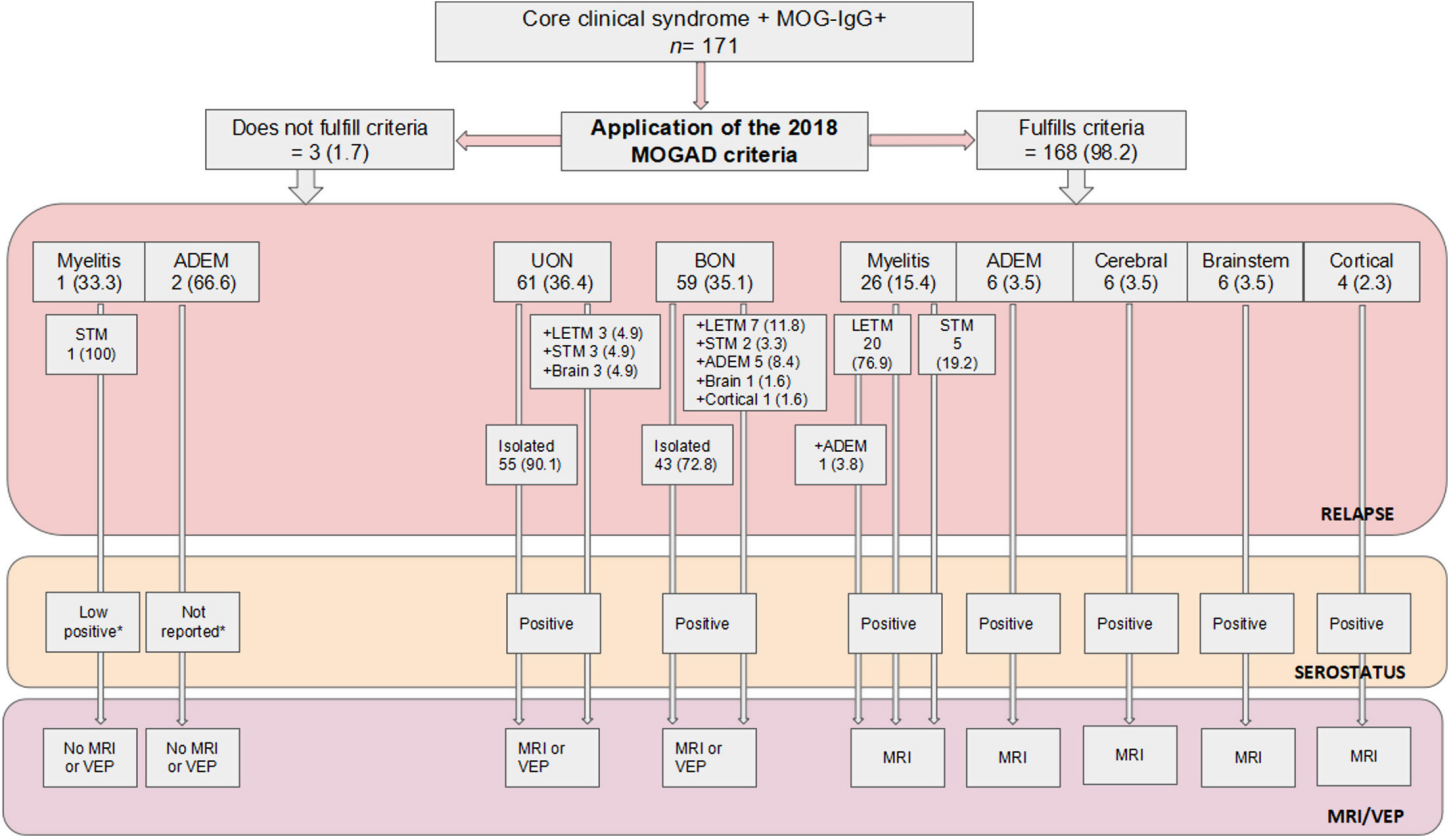
Application of 2018 myelin oligodendrocyte glycoprotein antibody (MOG‐IgG)‐associated disease (MOGAD) diagnostic criteria. All values are reported as absolute number and percentage. *In relation to MOG‐IgG titer. ADEM, acute disseminated encephalomyelitis; BON, bilateral optic neuritis; LETM, longitudinal extensive transverse myelitis; MRI, magnetic resonance imaging; STM, short transverse myelitis; UON, unilateral optic neuritis; VEP, visual evoked potentials.

Of 171 patients, 168 (98.2%) patients met the 2018 criteria. Of those who did not fulfill diagnostic criteria (*n* = 3), all tested positive for MOG‐IgG (one low‐positive and two with no reported titer), but none showed typical impairment in MRI or visual evoked potentials (VEPs; Figure 3).

#### Applicability of 2023 criteria

**FIGURE 4:**
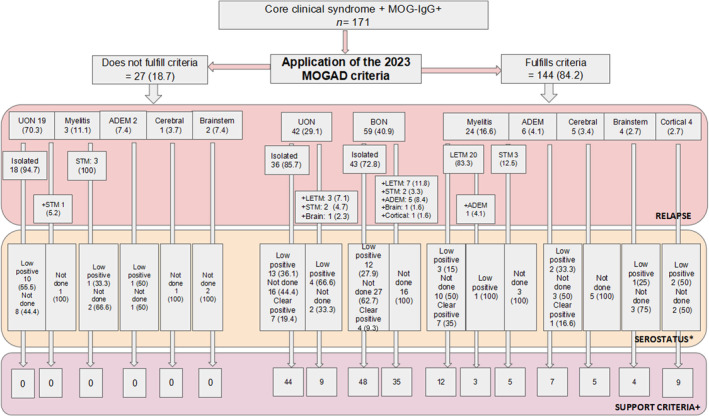
Application of 2023 myelin oligodendrocyte glycoprotein antibody (MOG‐IgG)‐associated disease (MOGAD) diagnostic criteria. All values are reported as absolute number and percentage. *In relation to MOG‐IgG titer. +A patient may have more than one supporting criteria. ADEM, acute disseminated encephalomyelitis; BON, bilateral optic neuritis; LETM, longitudinal extensive transverse myelitis; STM, short transverse myelitis; UON, unilateral optic neuritis.

Of 171 patients, 144 (84.2%) patients met the 2023 criteria, of whom 57 (39.5%) had MOG‐IgG+ titer information (19 clearly positive and 38 low‐positive), whereas 87 (60.5%) patients had no MOG‐IgG titer. All 144 patients had one or more diagnostic supporting criteria. The remaining 27 patients did not meet the 2023 MOGAD criteria due to low MOG‐IgG (*n* = 12) or lack of titer antibody access (*n* = 15), associated with absence of supporting criteria (Figure 4).

The 2023 MOGAD criteria showed a sensitivity of 86% (95% confidence interval [CI] = 0.80–0.91), specificity of 100% (95% CI 1–1), PPV of 100%, and NPV of 11% when compared to the 2018 criteria.

## DISCUSSION

In this retrospective cohort study, we have assessed the applicability of the 2023 MOGAD diagnostic criteria compared with 2018 criteria in real‐life settings in an LATAM population of 171 patients with at least one core clinical demyelinating event associated with MOG‐IgG+. We found that 98.2% and 84.2% of patients fulfilled the 2018 and the 2023 MOGAD diagnostic criteria, respectively.

MOG‐IgG titers play a fundamental role in the 2023 MOGAD criteria, and supporting criteria have been proposed to improve the specificity of this condition. However, MOG‐IgG testing as well as MOG‐IgG titers are not broadly available worldwide, especially in low‐income and limited‐resources countries like LATAM countries [4].

In our cohort of 144 patients meeting the 2023 MOGAD criteria, 60.2% did not have reported MOG‐IgG titers. In this context, the MOGAD diagnosis was established by meeting one or more diagnostic supporting criteria. Despite a significant number of patients lacking MOG‐IgG titer results, our diagnosis rate (84.2%) was in line with findings from US (81.5% [10] and 90% [11]) and Korean cohorts (93%) [9].

Among patients presenting as low‐positive or positive with no MOG‐IgG titers, 82.2% met the supporting criteria, consistent with results from both Korean (89%) [9] and US (80%) [11] cohorts. All clearly positive patients also fulfilled one or more supporting criteria, indicating a high sensitivity for MOG‐IgG in typical MOGAD presentations, as observed in other cohorts where the PPV of MOG‐IgG testing is titer dependent (PPVs: 1:1000, 100%; 1:100, 82%; 1: 20–40, 51%) [9, 10, 11, 15]. This validates the use of supporting criteria for patients with low‐positive or unknown MOG‐IgG titers.

Importantly, low‐positive MOG‐IgG titers in patients without supporting criteria should be interpreted with caution, as the PPV of MOG‐IgG has been reported as 10% (95% CI = 2%–40%) in those with atypical phenotypes and a titer < 1:100, and as 46% (95% CI = 33%–60%) in those with either atypical phenotypes or a titer < 1:100 [15]. These patients may actually be negative or atypical MOGAD patients who are being overlooked; perhaps additional supporting criteria could help resolve this situation [9, 15].

In this regard, in cases where cost is a concern for MOG‐IgG testing, it may be prudent to reserve titration testing for patients with atypical clinical features that do not match any of the supportive criteria, given that a clearly positive MOG‐IgG result could still suggest true MOGAD, even in an atypical presentation not previously associated with clear positivity in MOG‐IgG testing. This distinction is crucial for MOGAD, given the recent commercial availability of MOG‐IgG testing and the wide range of demographics, clinical presentations, disease courses, and treatment responses linked to this entity [16]. In this context, we found that 15.7% of our patients did not meet the 2023 MOGAD criteria, as none of them satisfied any supportive criteria (clinical and neuroradiological assessments yielded negative results), half experienced low‐positive MOG‐IgG, and the remaining half lacked access to MOG‐IgG titration; in these cases, a clear MOG‐IgG+ result from titration could have led to MOGAD diagnosis, although as previously mentioned, it would have been an atypical presentation. Additionally, recent comparative studies analyzing MOG‐IgG detection methods have shown that live CBA may be more sensitive than fixed CBA [17]. However, two recent studies involving 322 and 257 patient samples demonstrated excellent agreement between live and fixed CBA for diagnosing MOGAD [18, 19].

As a result of both findings (high MOG‐IgG sensitivity in typical MOGAD presentations and the absence of clearly positive MOG‐IgG in atypical patients), a debate should arise. Are these atypical cases truly indicative of an atypical MOGAD, or are we potentially observing other conditions where the presence of MOG‐IgG is merely a secondary phenomenon or a result of cross‐immunogenicity, as described in MS [20]? This hypothesis is based on the finding that, despite the increasing knowledge of clinical MOGAD presentations, the pathophysiology and importantly, the pathogenic role of human MOG‐IgG remain to be fully determined; given that this antibody has a complex and dynamic epitope specificity [21], and based on the observation that even with effective B‐cell depletion treatments using anti‐CD20 medications, only 55% of patients remained free from relapses in the first year and 33% in the second year, B cells may not be the sole cell type involved in MOGAD pathophysiology [21, 22, 23]. On the other hand, because MOG‐IgG sensitivity has been proven to be high in typical cases (100% of patients in our cohort as well as other series have one or more supporting criteria) [9], a possibility in regions like LATAM countries might be to rest MOGAD diagnosis on supporting criteria and MOG‐IgG positivity only (without titers), as atypical cases do not present clearly positive MOG‐IgG tests in other cohorts [4, 9, 10, 11]. For instance, in our cohort, if patients had absence of MOG‐IgG titers, diagnostic rates would have been the same, as ultimately, MOGAD diagnosis was based on the presence of supporting criteria, highlighting clinical and MRI findings for MOGAD.

When comparing the 2018 criteria with those of 2023, diagnostic rate decreased by 14% (from 98.2% in 2018 to 84.2% in 2023), mainly because almost 75% of undiagnosed patients according to the 2023 criteria presented as unilateral ON, approximately half of them had low MOG‐IgG titers, and the other half had no available titration tests, thus not meeting the 2023 MOGAD antibody criteria or any required supporting criteria, as illustrated in Figure 4. In this context, when MOG‐IgG titers are not available, two questions may also arise. What is the impact of supporting criteria in clinical practice? Could VEPs, previously used in 2018, regain value in this clinical scenario? In our cohort, 19 patients with unilateral ON did not meet the 2023 MOGAD criteria, of whom 73.6% (*n* = 14) exhibited pathological VEPs; including VEPs in the supportive criteria would have meant an increase in diagnostic rate from 84.2% to 95.9%. The 2023 MOGAD criteria showed a high sensitivity (86%), specificity (100%), and PPV (100%), but a low NPV (11%) was observed when compared to the 2018 criteria. In this regard, a US study has shown a sensitivity of 100% and specificity of 55% for the 2023 MOGAD criteria, although methodological differences in sensitivity and specificity definition and comparisons were detected [10, 11, 12]. Another beneficial evaluation to consider may be optical coherence tomography measurements of the peripapillary retinal nerve fiber layer thickness, as they have shown higher values in acute MOGAD‐ON compared to MS due to optic disc edema, thus offering greater specificity [21, 24].

Given that the optic nerve head vulnerability is likely due to a lack of microvessels with blood–brain barrier characteristics and nonspecific permeability [21, 25, 26, 27], low MOG‐IgG titers may be sufficient to trigger an immune response in this anatomic region, leading to local compromise of oligodendrocytes. Unfortunately, MOG‐IgG detection in CSF is not present in a high proportion of cases, as reported in a previous study [20], where CSF MOG‐IgG was undetectable in most patients with isolated ON. The unidirectional flow of CSF from the intracranial subarachnoid space (SAS) to the orbital SAS [28] may account for the lack of antibody detection in patients sampled through lumbar puncture [20].The presence of CSF MOG‐IgG testing in these undiagnosed ON patients probably would not have changed the diagnostic rate in our cohort. However, paired serum and CSF MOG‐IgG positivity was found in 56.8% of MOGAD patients from an international multicenter study and was linked to a more severe clinical presentation [27]. CSF‐only MOG‐IgG positivity can manifest in patients with a phenotype indicative of MOGAD and is linked to poorer outcome. These results suggest clinical significance in evaluating CSF MOG‐IgG in patients with a phenotype of MOGAD, irrespective of the MOG‐IgG serostatus.

It is yet to be determined whether patients without MOGAD‐specific clinical and imaging findings are atypical MOGAD or they present other unclassified CNS inflammatory demyelinating disease [9, 29, 30]. Given that apart from high MOG‐IgG titer by live CBA, MOGAD lacks other specific serological or radiological biomarkers, initial validation studies of the new 2023 criteria will need to rely on clinical judgment as a comparator when assessing patients with low titers or suboptimal testing methodology [12]. In this context, red flag findings may be developed to better discriminate TN patients. Thus, the use of conventional MRI to identify supporting criteria or typical MOGAD lesions and MRI findings observed in diseases other than MOGAD may help to improve the specificity and sensitivity. Likewise, MRI criteria have been established to correctly distinguish MS from NMOSD and MOGAD, the main differential diagnoses, in diverse populations with high accuracy, including LATAM populations [31]. However, these criteria have not proven effective in distinguishing MOGAD from NMOSD. A recent LATAM study showed that chiasmatic lesions (31.7%) were more frequent in NMOSD‐ON than MOGAD‐ON patients (13.1%, *p* = 0.01), whereas orbital (anterior) optic nerve lesions (14%) were more prevalent in MOGAD‐ON compared with NMOSD‐ON patients (*p* < 0.001) [32].

Given that nine of 11 supporting criteria are based on MRI findings and having just one of them may significantly impact the diagnosis, it would be beneficial to have international standardized spinal cord, brain, and orbit MRI protocols to evaluate the detection of supportive criteria before proceeding with specific MOG‐IgG testing (titration vs. no titration) if necessary. An interesting observation from our cohort is that under the 2018 criteria, two patients were not diagnosed despite presenting with ADEM‐like symptoms and positive antibodies (titers not specified) due to the lack of access to MRI and VEPs. This highlights how complementary examinations, such as MRI, can influence diagnosis, emphasizing the need for standardized MRI protocols. A similar situation was noted when investigating perineural optic enhancement, which was the most common supporting criterion related to ON in our cohort (30.8%) after bilateral ON (36.4%), but its identification was lower than reported in other cohorts (50%–88%) [21]. Perhaps an adequate technique (including fat saturation sequences) or gadolinium dose could have helped in finding this MRI abnormality, and therefore, establishing MOGAD diagnosis. In this study, disc edema was found in a small proportion of patients. This could be due to the study's design (lack of standardized ophthalmological assessments). Thus, the prevalence of supporting criteria in ON may suggest that not all criteria have the same specificity for MOGAD, which is yet to be determined in future studies.

This study has several limitations that need to be addressed. The retrospective design, with a relatively small sample size limited to adult patients, and the exploratory nature of the study are the main limitations; therefore, findings should be carefully interpreted. However, prevalence of MOG‐IgG+ in AQP4‐IgG− patients and availability of MOG‐IgG testing are lower in LATAM countries [8, 33]. Comparing the performance of the 2023 MOGAD criteria between adults and children was not feasible in this study. Additionally, unintentional selection and referral bias may have occurred, influencing patient characteristics and results. We applied as the “gold standard” the “not formal” 2018 criteria, which were the only ones available, until the description of these new MOGAD criteria, for comparing the performance of the 2023 MOGAD criteria, and only evaluated consecutive patients with clinical core demyelinating events plus MOG‐IgG+; thus, the included patients impact specificity, consequently influencing the results. In this context, our study did not involve patients with a diagnosis other than MOGAD and thus is not appropriate to fully and formally validate the performance of the 2023 MOGAD criteria. Exclusion of alternative diagnoses was not standardized in the entire cohort, depending on clinical judgment. Live or fixed CBA serum testing determination was not always known; irregular intervals, titers, and timing, the absence of CSF determinations (although it can be helpful in adult cases) [34], and noncentralized determinations were other relevant limitations. These aspects can influence antibody results and titers and therefore the performance of the tested criteria. Additionally, there was no standardized protocol for the assessment of clinical and MRI findings. Of note, these results represent real‐world evidence of daily clinical practice in a realistic LATAM context.

In conclusion, our findings support the diagnostic utility of the 2023 MOGAD criteria in a real‐world cohort, despite the limited access to MOG‐IgG titration. We have found a good performance of the 2023 MOGAD diagnostic criteria in consecutive LATAM patients with clinical core demyelinating events plus MOG‐IgG+ tested by CBA. These results also contribute to the international dataset for comparison with previously published results from Asia, North America, and Europe. Our results emphasize the significance of assays and supporting criteria in patient diagnoses and the crucial role of proper assessment in these patients.

## AUTHOR CONTRIBUTIONS

**Edgar Carnero Contentti:** Conceptualization; investigation; writing – original draft; methodology; validation; visualization; formal analysis; data curation; supervision; project administration; resources; writing – review and editing. **Claudia Pestchanker:** Conceptualization; investigation; writing – original draft; methodology; validation; visualization; writing – review and editing; formal analysis; project administration; data curation; supervision; resources. **Ethel Ciampi:** Investigation; writing – review and editing; data curation. **Sheila Castro Suarez:** Data curation; writing – review and editing; investigation. **Cesar Caparo Zamalloa:** Investigation; writing – review and editing; data curation. **Vanesa Daccach Marques:** Investigation; writing – review and editing; data curation. **Katharina Messias:** Conceptualization; writing – review and editing; data curation. **José Ignacio Gortari:** Conceptualization; writing – review and editing; data curation. **Verónica Tkachuk:** Conceptualization; writing – review and editing; data curation. **Berenice Silva:** Conceptualization; writing – review and editing; data curation. **Carolina Mainella:** Investigation; writing – review and editing; data curation. **Saúl Reyes:** Investigation; writing – review and editing; data curation. **Jaime Toro:** Conceptualization; writing – review and editing; data curation. **Juan Rodriguez:** Conceptualization; writing – review and editing; data curation. **Edgar Correa‐Diaz:** Conceptualization; writing – review and editing; data curation. **Juan I. Rojas:** Investigation; validation; visualization; writing – review and editing; data curation. **Friedemann Paul:** Investigation; writing – original draft; methodology; writing – review and editing; supervision.

## CONFLICT OF INTEREST STATEMENT

None of the authors has any conflict of interest to disclose.

## Data Availability

The data that support the findings of this study are available from the corresponding author upon reasonable request.
